# Correction: Engineered migrasomes provide a robust and thermally stable vaccination platform

**DOI:** 10.7554/eLife.113497

**Published:** 2026-10-16

**Authors:** Dongju Wang, Haifang Wang, Wei Wan, Zihui Zhu, Takami Sho, Yi Zheng, Xing Zhang, Longyu Dou, Qiang Ding, Li Yu, Zhihua Liu

**Keywords:** Mouse

Wang D, Wang H, Wan W, Zhu Z, Sho T, Zheng Y, Zhang X, Dou L, Ding Q, Yu L, Liu Z. 2025. Engineered migrasomes provide a robust and thermally stable vaccination platform. *eLife* **13**:RP97621. doi: 10.7554/eLife.97621.

Published 13 November 2025

We were notified via PubPeer of a duplication of four data points in the source data corresponding to Figure 2C and Figure 2D. Upon reviewing the source data, we identified that Figure 2C contains four inadvertently duplicated data points. The duplication arose during figure assembly: we used the same GraphPad template to generate the statistical plots for Figures 2C and 2D in order to maintain a consistent presentation style, and the Figure 2D data had not been fully cleared from the template before the Figure 2C data were entered. This is purely a copy-and-paste error during figure preparation; it does not reflect any issue with the underlying experimental measurements.

After carefully reviewing the original source data, we confirmed that these four duplicated data points should not be included in the Figure 2C dataset. The correct sample size for Figure 2C is therefore N=40, rather than N=44. We confirmed that the data in Figure 2D are correct and that Figure 2D is unaffected by this error; the duplication was introduced only during the assembly of Figure 2C. We have reanalyzed the corrected dataset and confirmed that removing these four duplicated data points does not change the statistical significance of the comparison and does not affect the experimental results or the conclusions of our study. The error was limited to the assembly of the figure and did not arise from the underlying experimental measurements.

The corrected Figure 2 (updated for panel C) is shown here:

**Figure 1:**
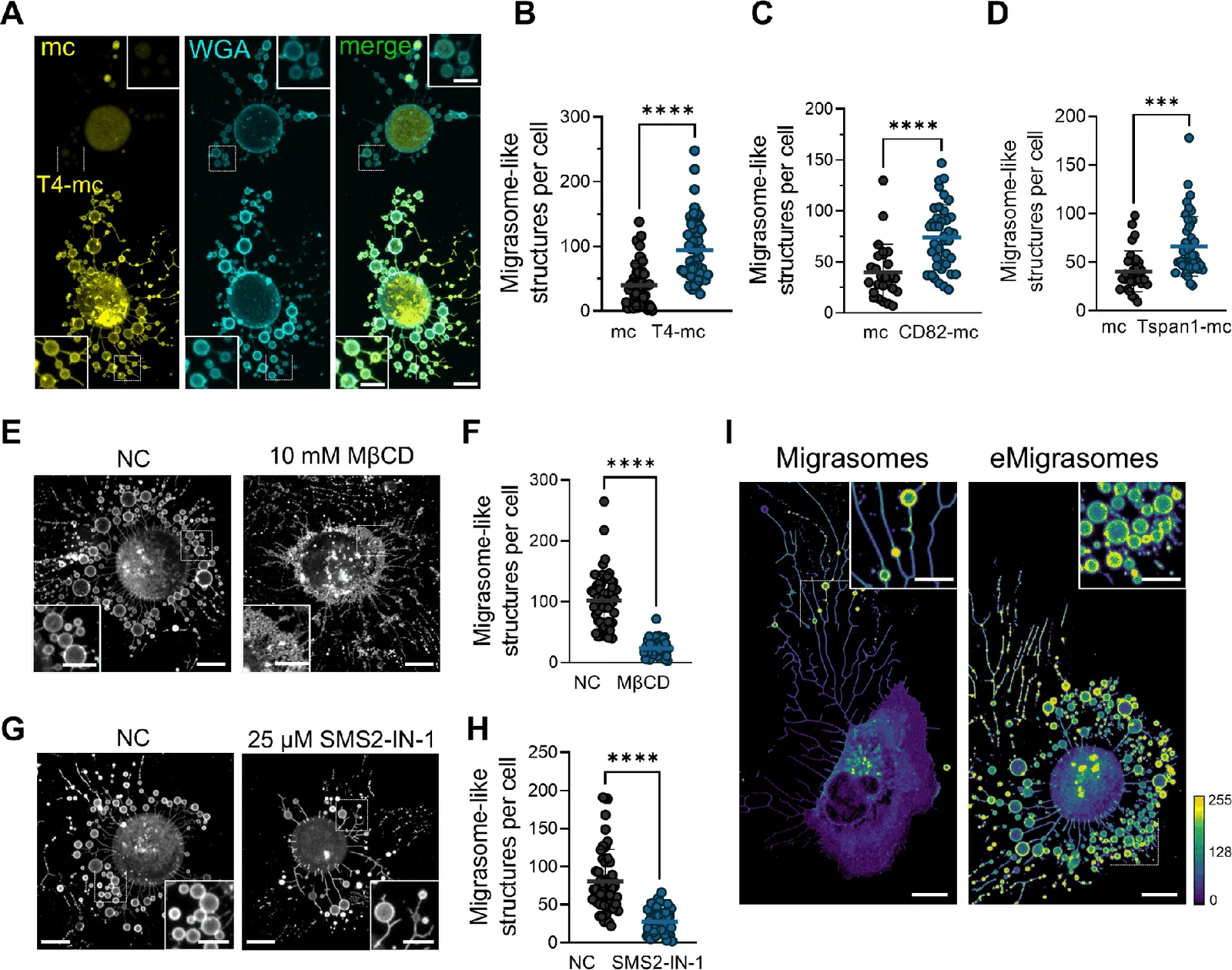


The originally published Figure 2 is shown for reference:

**Figure 2:**
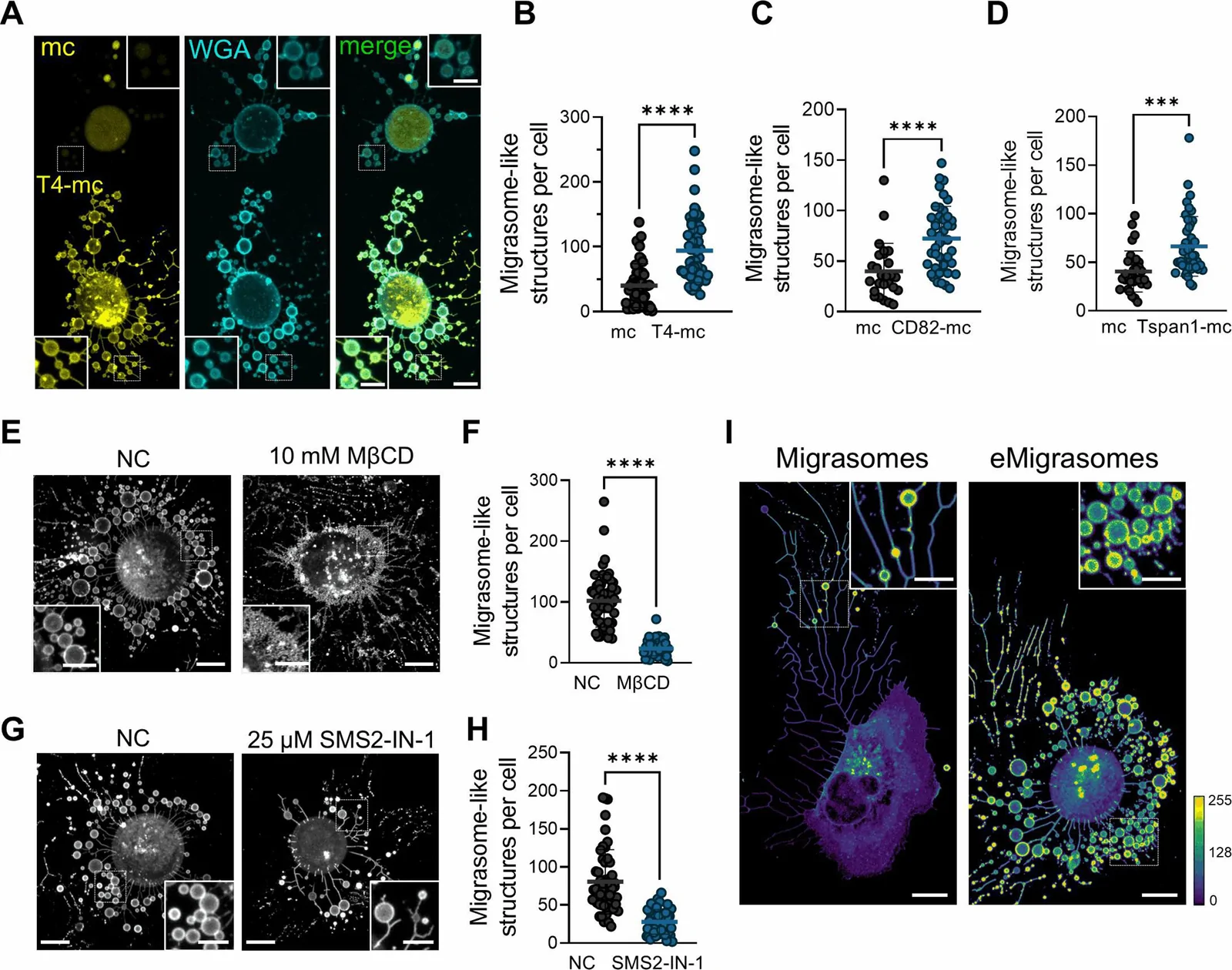


The corrected Figure 2C legend is shown here (underline to highlight changes):

(C) Statistical analysis of the number of migrasome-like structures per cell in NRK cells transiently transfected with mCherry vector (n=26 cells) or CD82-mCherry (n=40 cells) in Figure 2—figure supplement 1A. Data were plotted as mean ± SD.

The originally published Figure 2C legend is shown here:

(C) Statistical analysis of the number of migrasome-like structures per cell in NRK cells transiently transfected with mCherry vector (n=26 cells) or CD82-mCherry (n=44 cells) in Figure 2—figure supplement 1A. Data were plotted as mean ± SD.

We have also corrected the underlying Source data for this figure (Figure 2—source data 1).

The article has been corrected accordingly.

