# Supplementary material for: Engineered migrasomes provide a robust and thermally stable vaccination platform
Source: eLife. 2025 Nov 13;13:RP97621. doi: 10.7554/eLife.97621 (PMC12614892; doi:10.7554/eLife.97621)
Supplement: Figure 3—source data 1. [file elife-97621-fig3-data1.zip › Figure 3- source data 1.pdf]

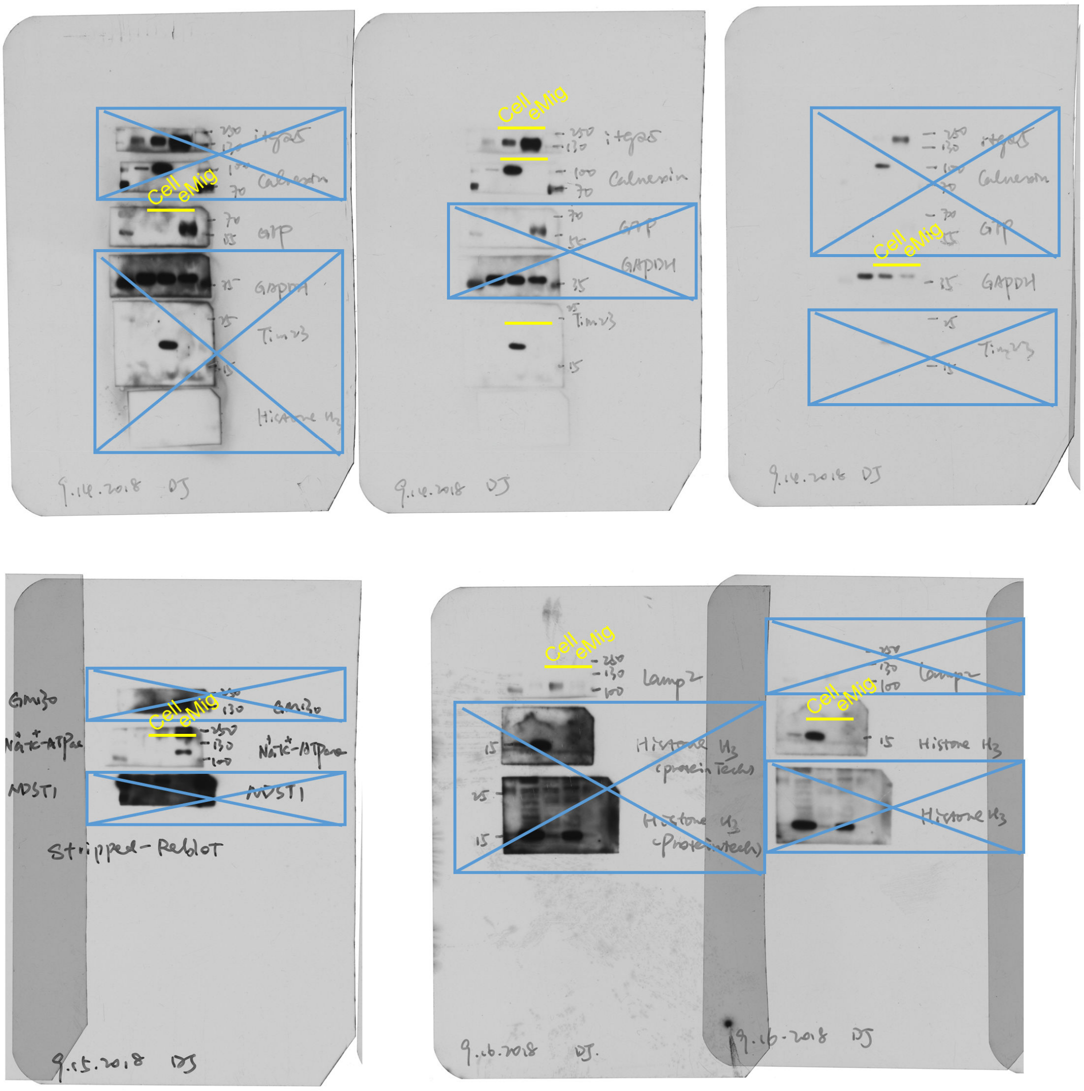

Figure 3, Source Data 1. Original films corresponding to Figure 3F. Rainbow molecular weight markers were employed and labeled. The corresponding primary antibodies were also labeled.
