# Supplementary figures and images for: Engineered migrasomes provide a robust and thermally stable vaccination platform
Source: eLife. 2025 Nov 13;13:RP97621. doi: 10.7554/eLife.97621 (PMC12614892; doi:10.7554/eLife.97621)

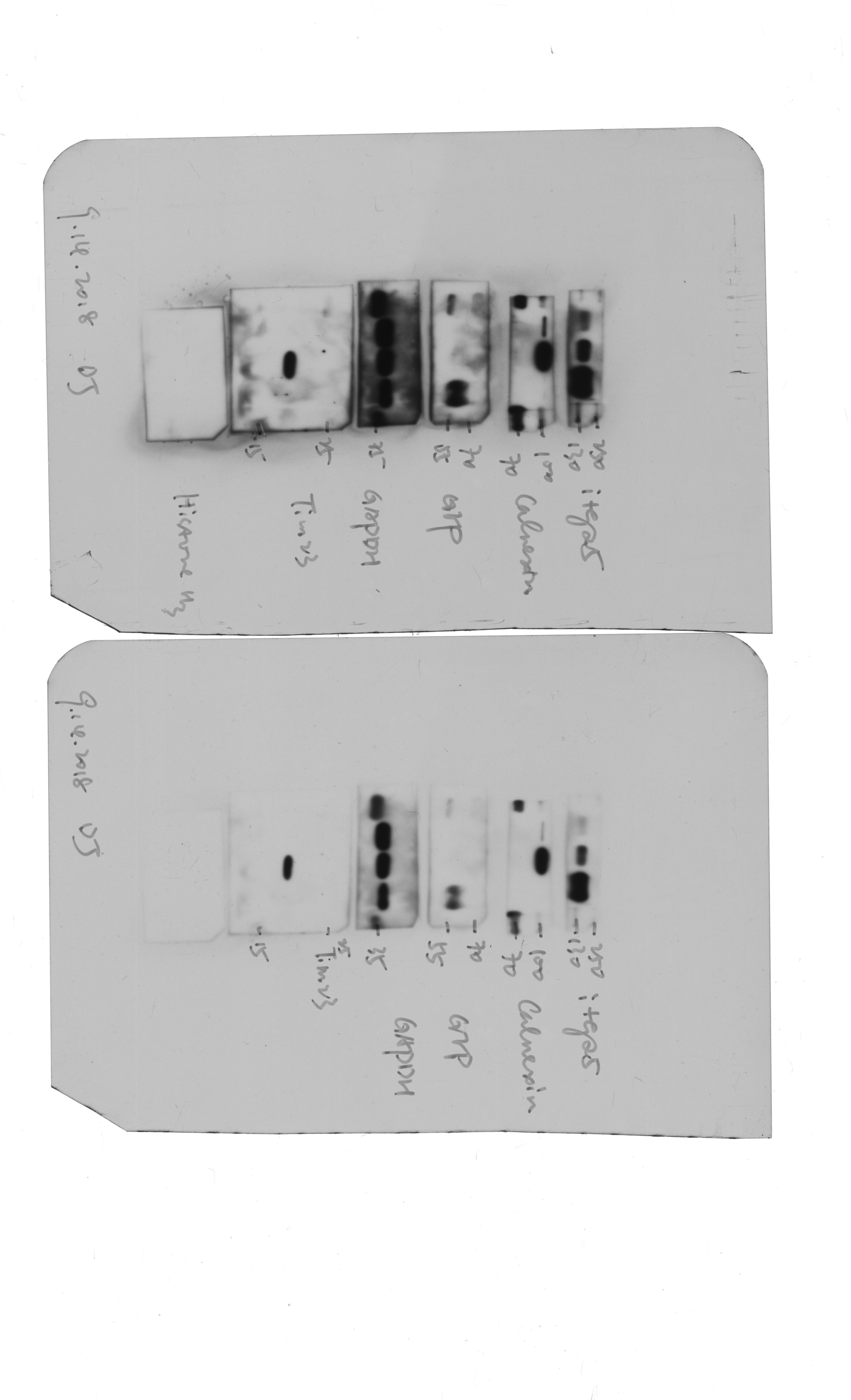

Supplement: Figure 3—source data 2. [file elife-97621-fig3-data2.zip › Figure 3-source data 2/1005.tif]

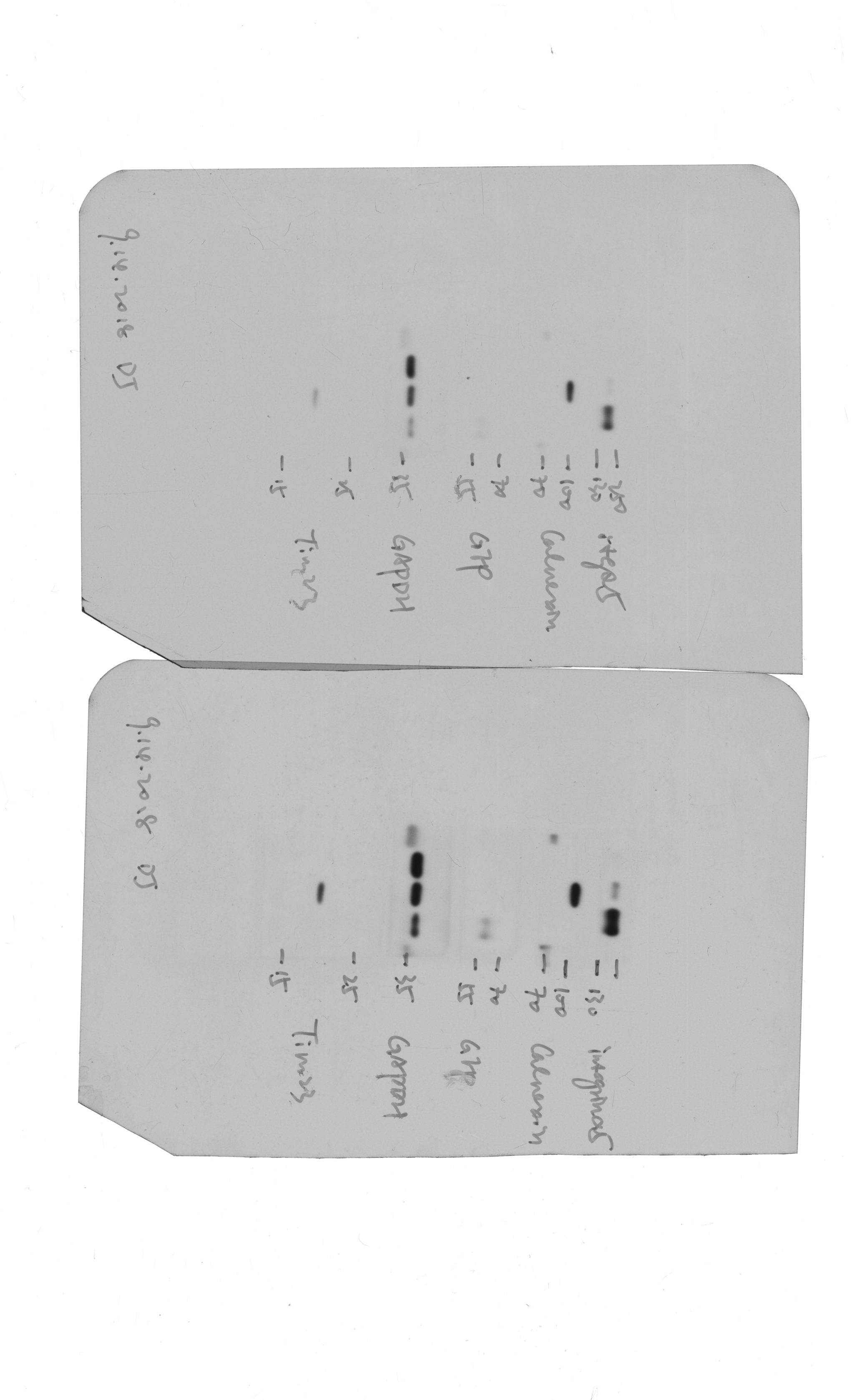

Supplement: Figure 3—source data 2. [file elife-97621-fig3-data2.zip › Figure 3-source data 2/1006.tif]

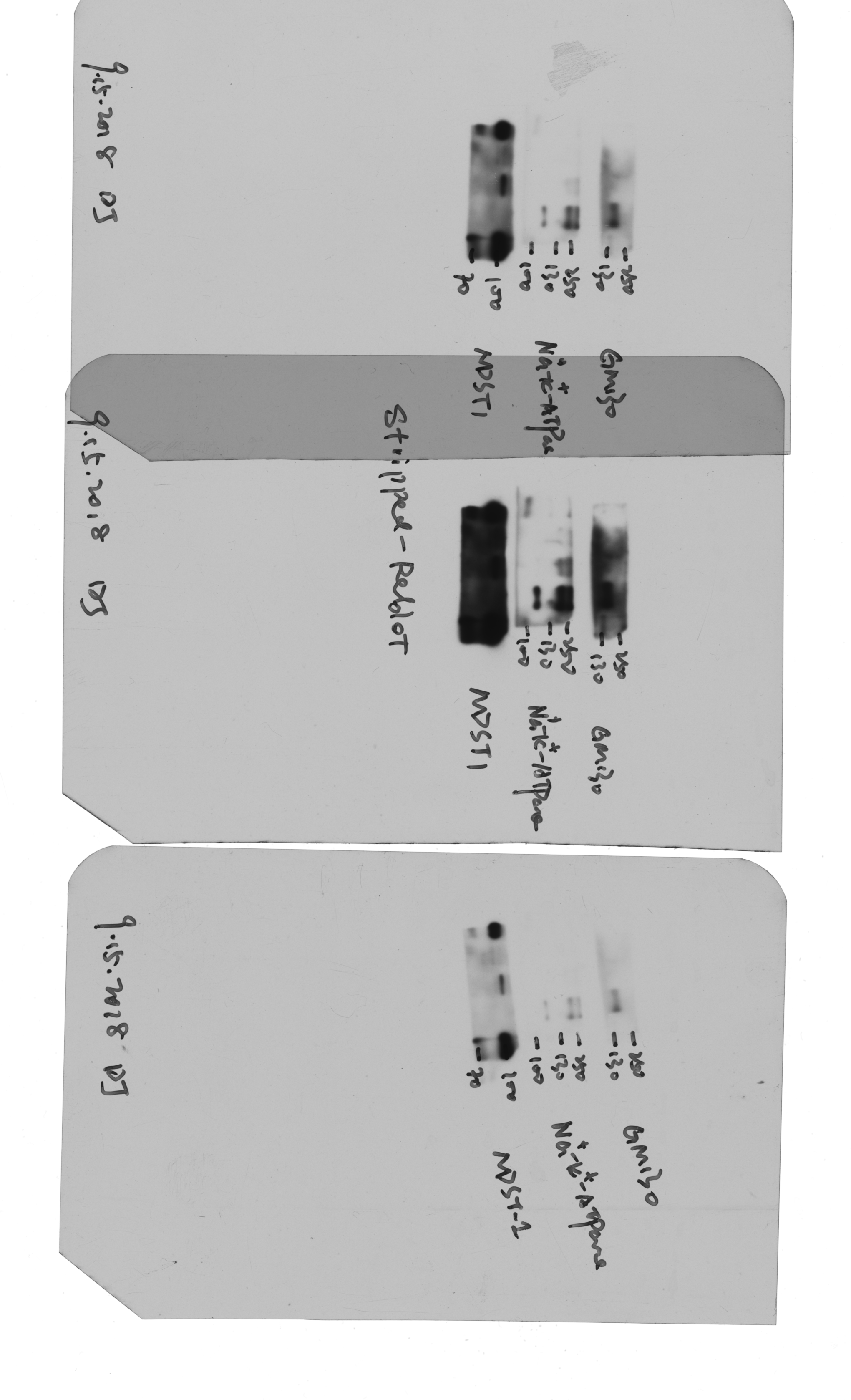

Supplement: Figure 3—source data 2. [file elife-97621-fig3-data2.zip › Figure 3-source data 2/1008.tif]

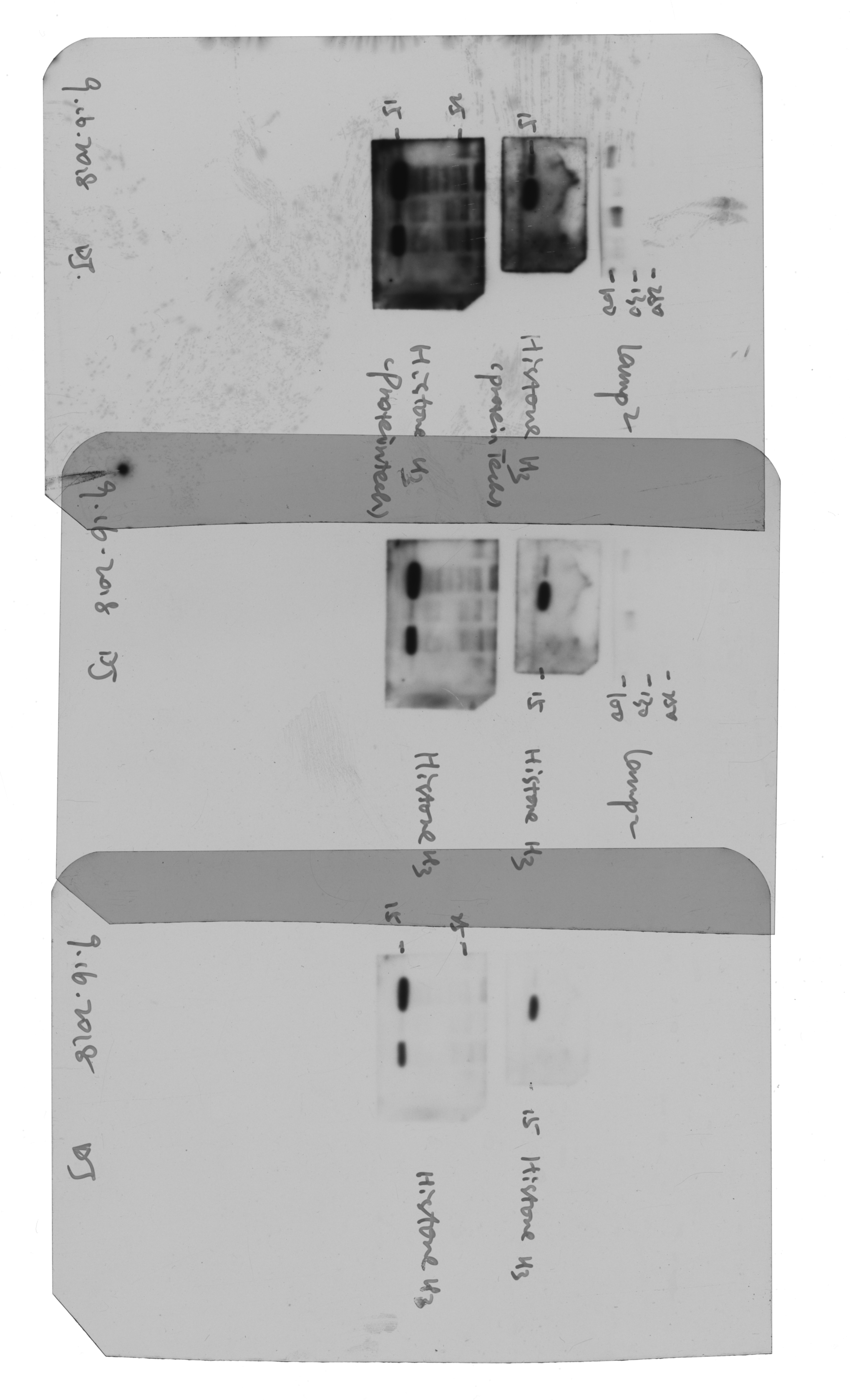

Supplement: Figure 3—source data 2. [file elife-97621-fig3-data2.zip › Figure 3-source data 2/1009.tif]

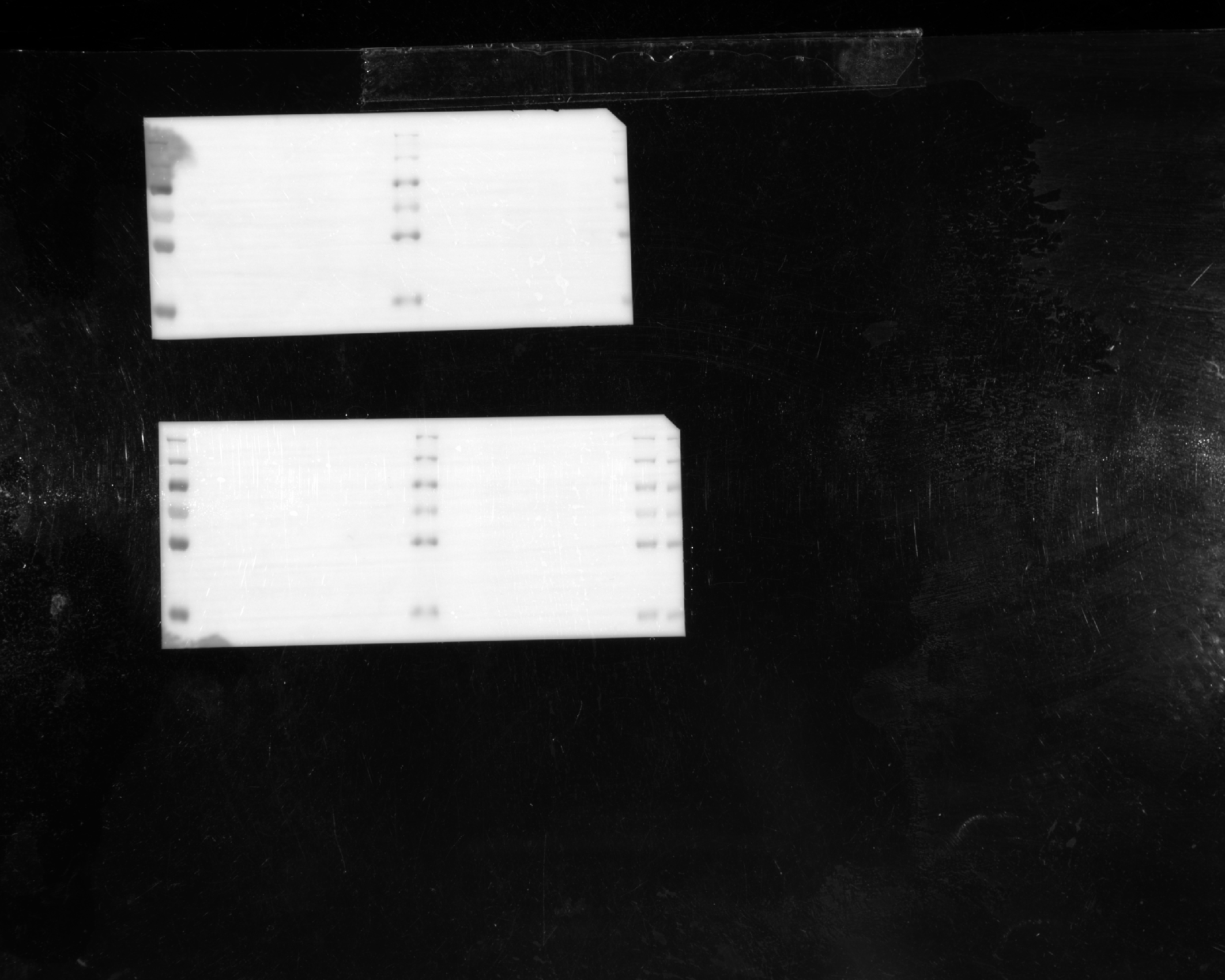

Supplement: Figure 4—source data 2. [file elife-97621-fig4-data2.zip › Figure 4-source data 2/yuli lab 2022-08-04 0.351s 10.666s(Colorimetric).tif]

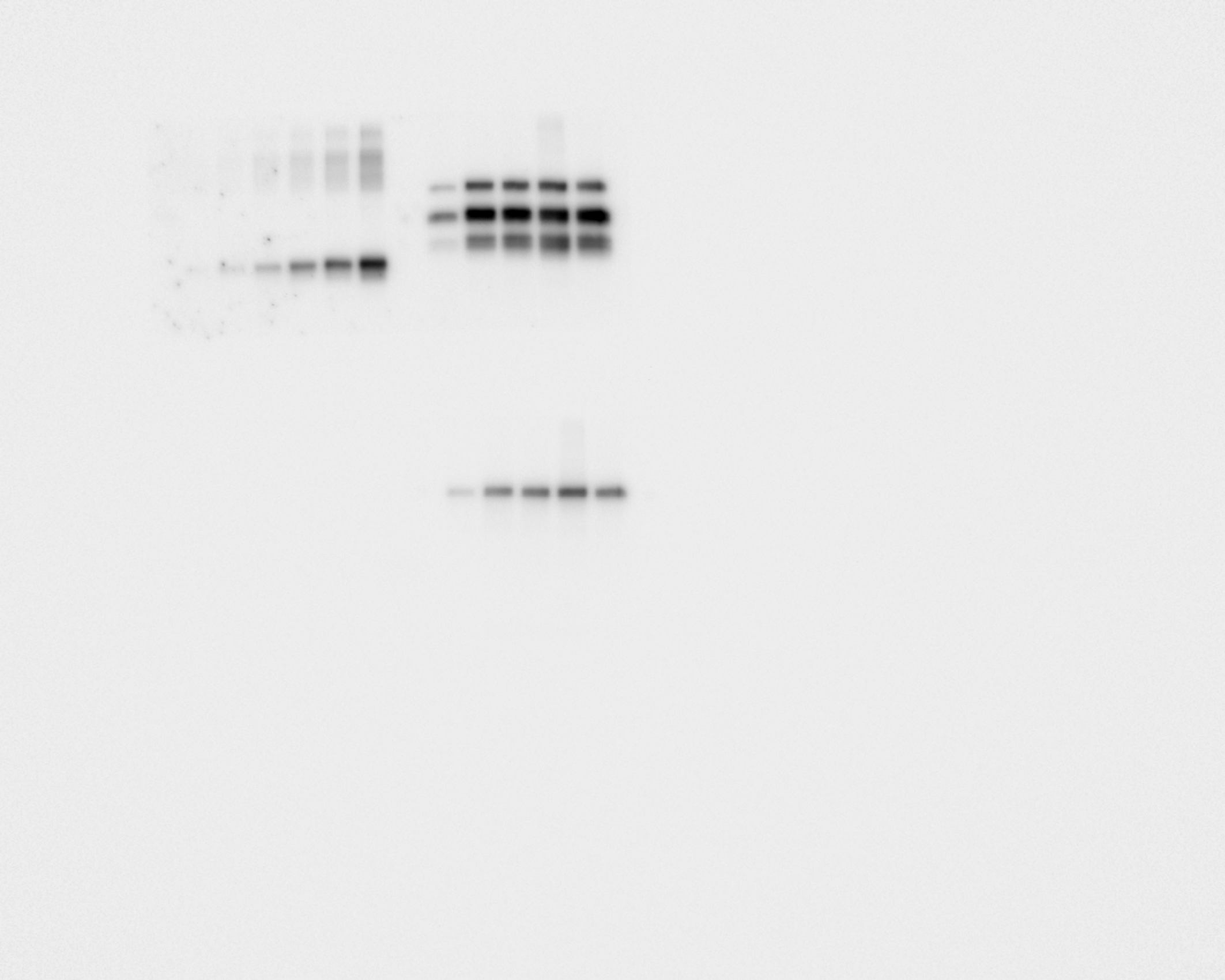

Supplement: Figure 4—source data 2. [file elife-97621-fig4-data2.zip › Figure 4-source data 2/yuli lab 2022-08-04 10.666s(Chemiluminescence).tif]

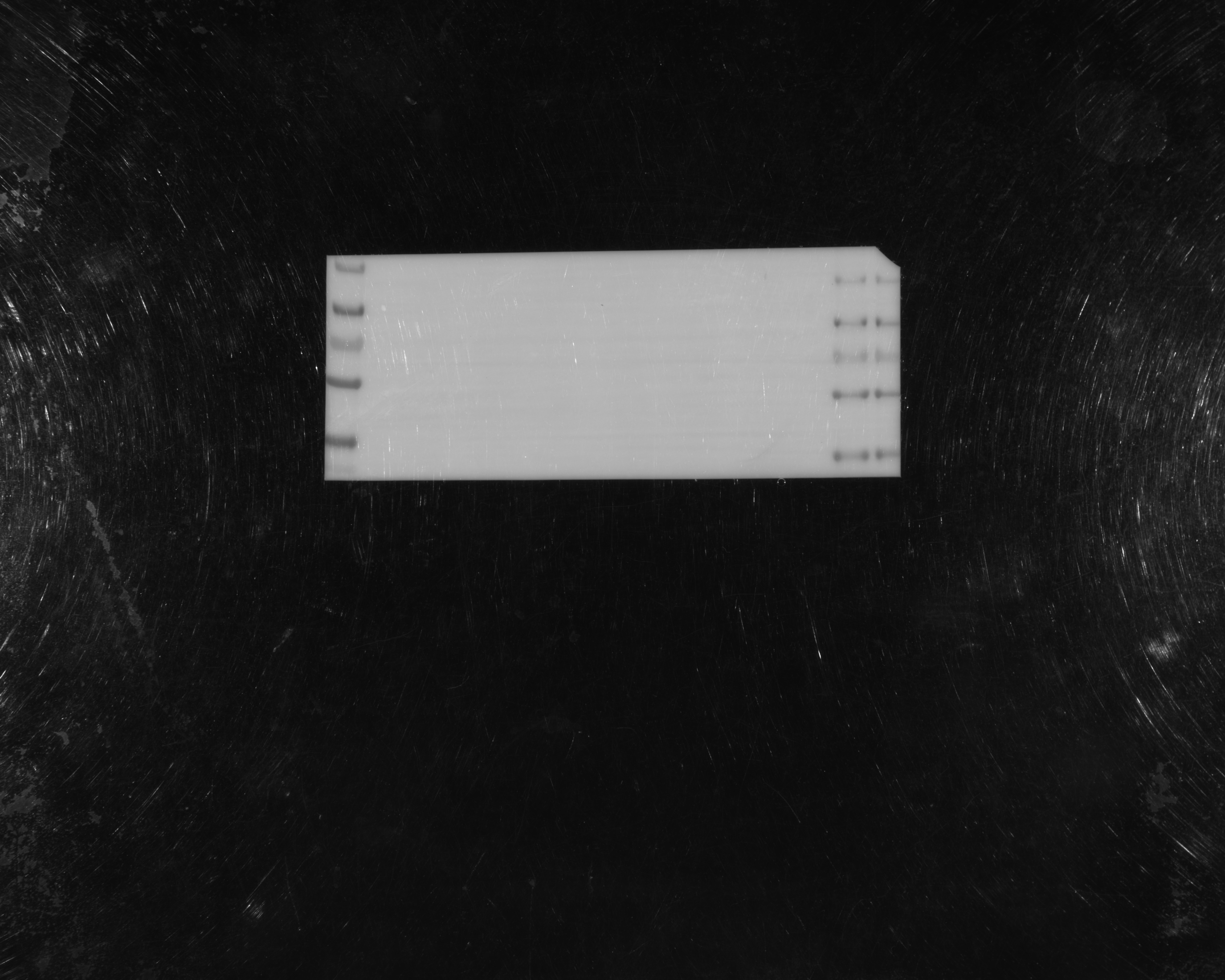

Supplement: Figure 4—source data 2. [file elife-97621-fig4-data2.zip › Figure 4-source data 2/yuli lab 2023-06-30 0.753s(Colorimetric).raw16.tif]

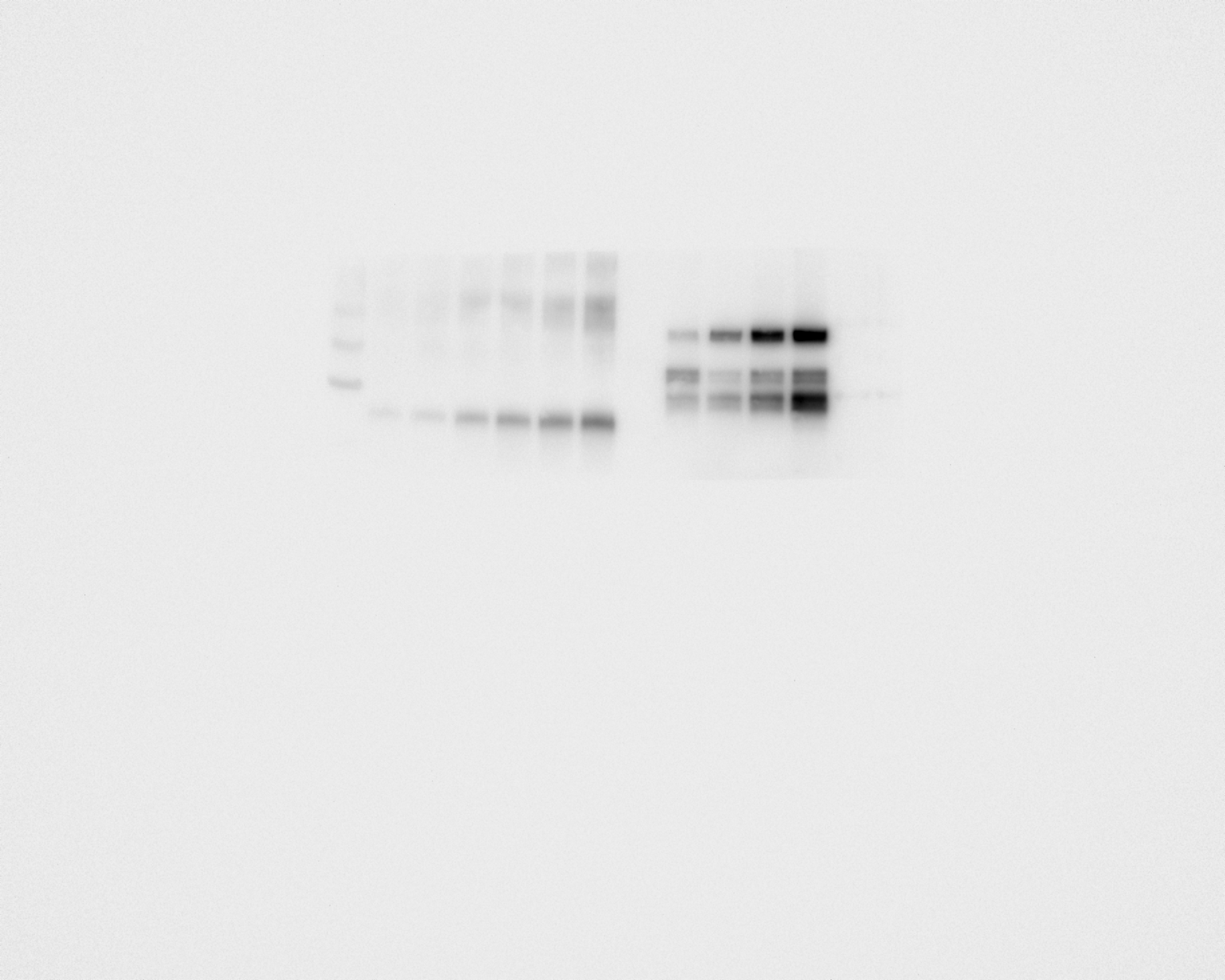

Supplement: Figure 4—source data 2. [file elife-97621-fig4-data2.zip › Figure 4-source data 2/yuli lab 2023-06-30 299.998s(Chemiluminescence).tif]

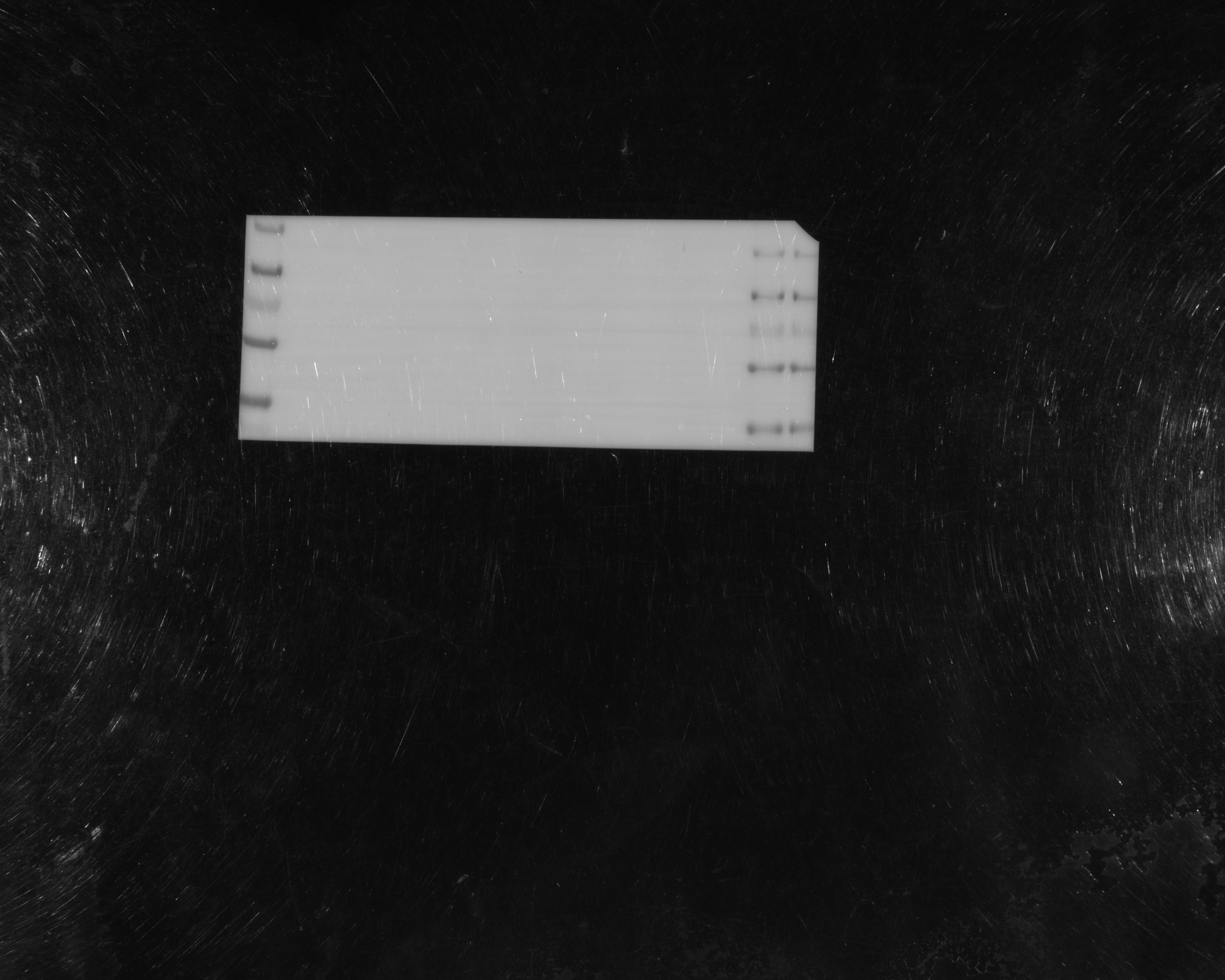

Supplement: Figure 4—source data 2. [file elife-97621-fig4-data2.zip › Figure 4-source data 2/yuli lab 2023-07-02 0.753s 179.992s(Colorimetric).raw16.tif]

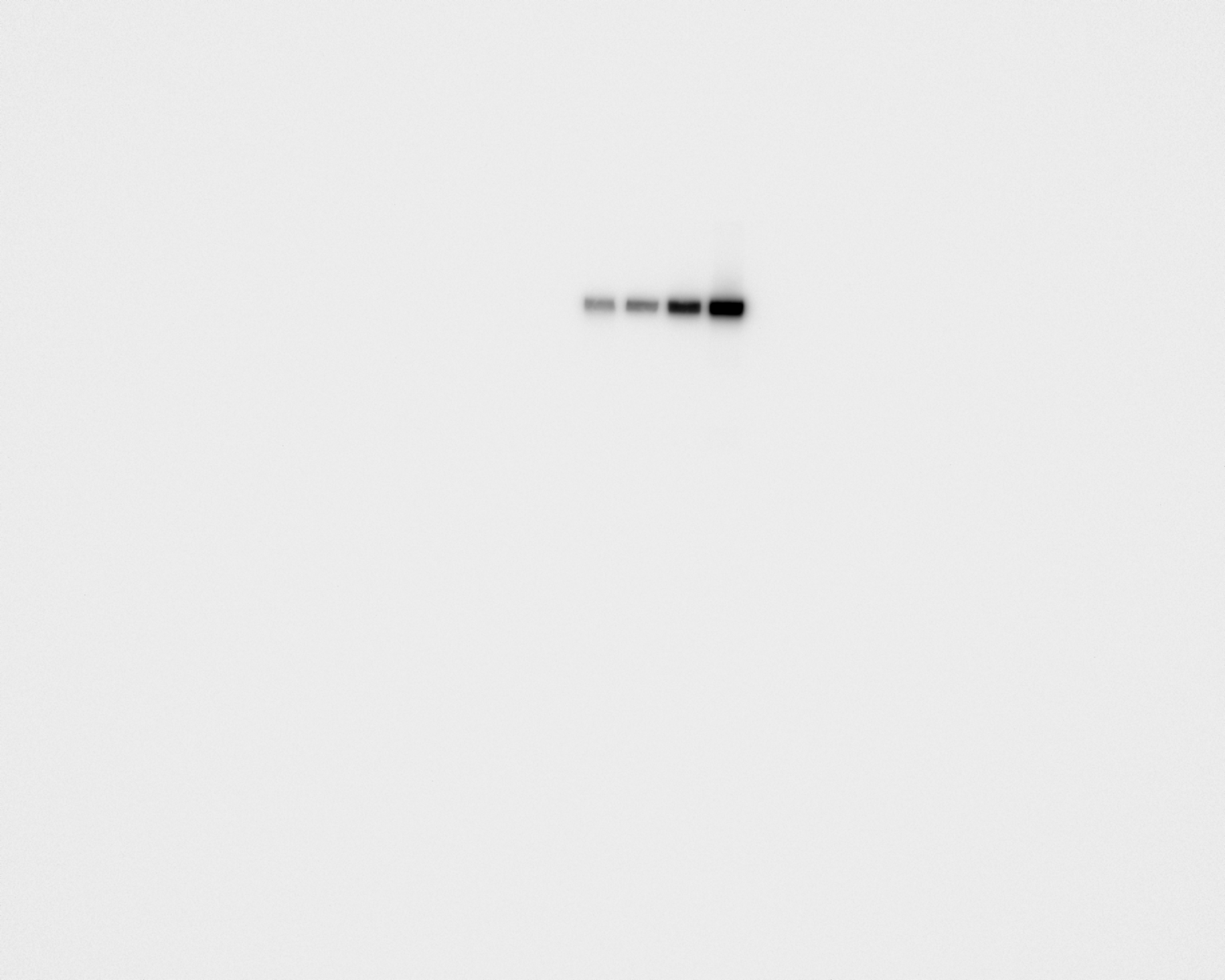

Supplement: Figure 4—source data 2. [file elife-97621-fig4-data2.zip › Figure 4-source data 2/yuli lab 2023-07-02 160.104s(Chemiluminescence).tif]

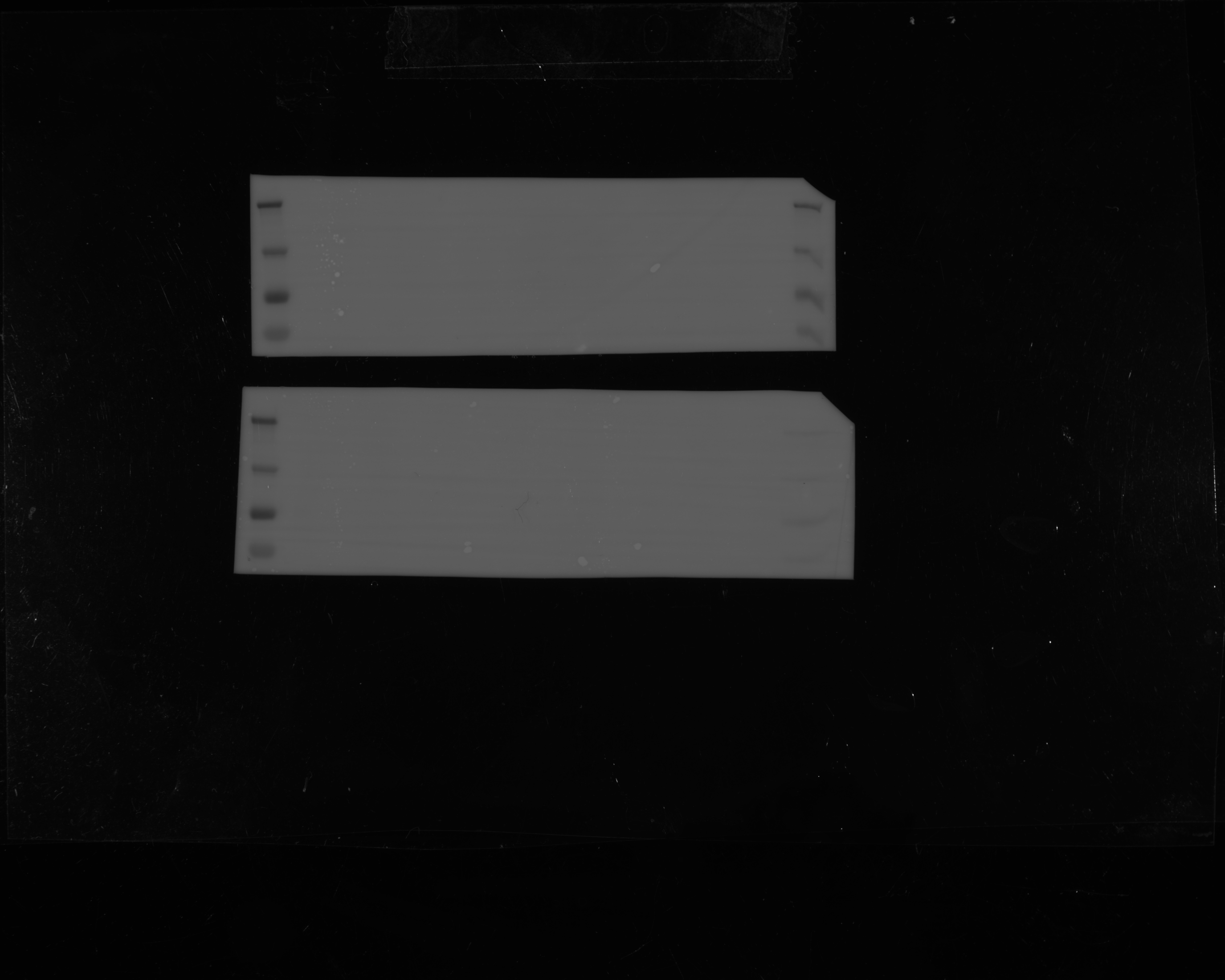

Supplement: Figure 5—source data 2. [file elife-97621-fig5-data2.zip › Figure 5-source data 2/yuli lab 2021-01-29 0.372s 9.500s(Colorimetric).raw16.tif]

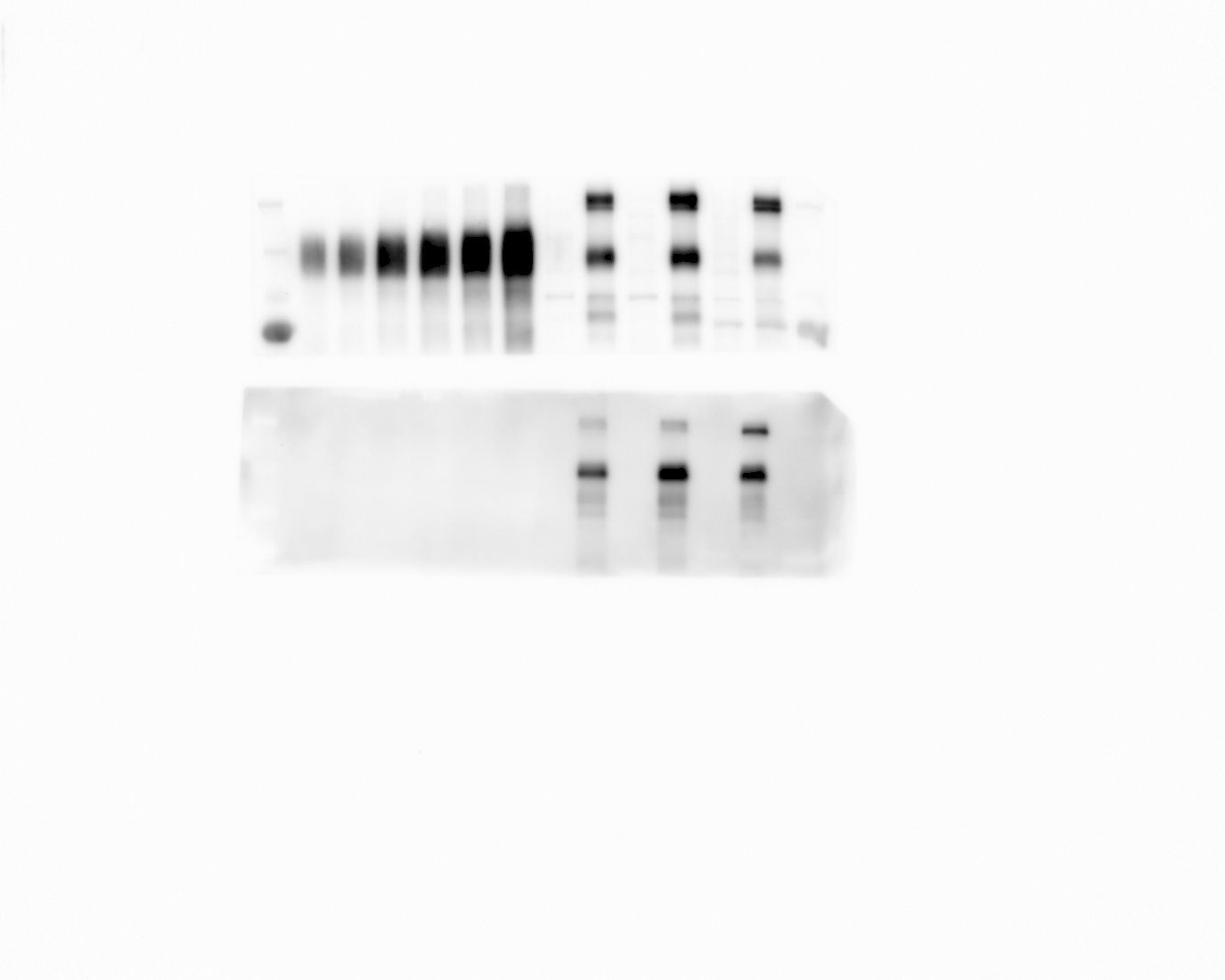

Supplement: Figure 5—source data 2. [file elife-97621-fig5-data2.zip › Figure 5-source data 2/yuli lab 2021-01-29 60.500s(Chemiluminescence).raw16.tif]
