# Supplementary material for: Engineered migrasomes provide a robust and thermally stable vaccination platform
Source: eLife. 2025 Nov 13;13:RP97621. doi: 10.7554/eLife.97621 (PMC12614892; doi:10.7554/eLife.97621)
Supplement: Figure 4—source data 1. [file elife-97621-fig4-data1.zip › Figure 4-source data 1.pdf]

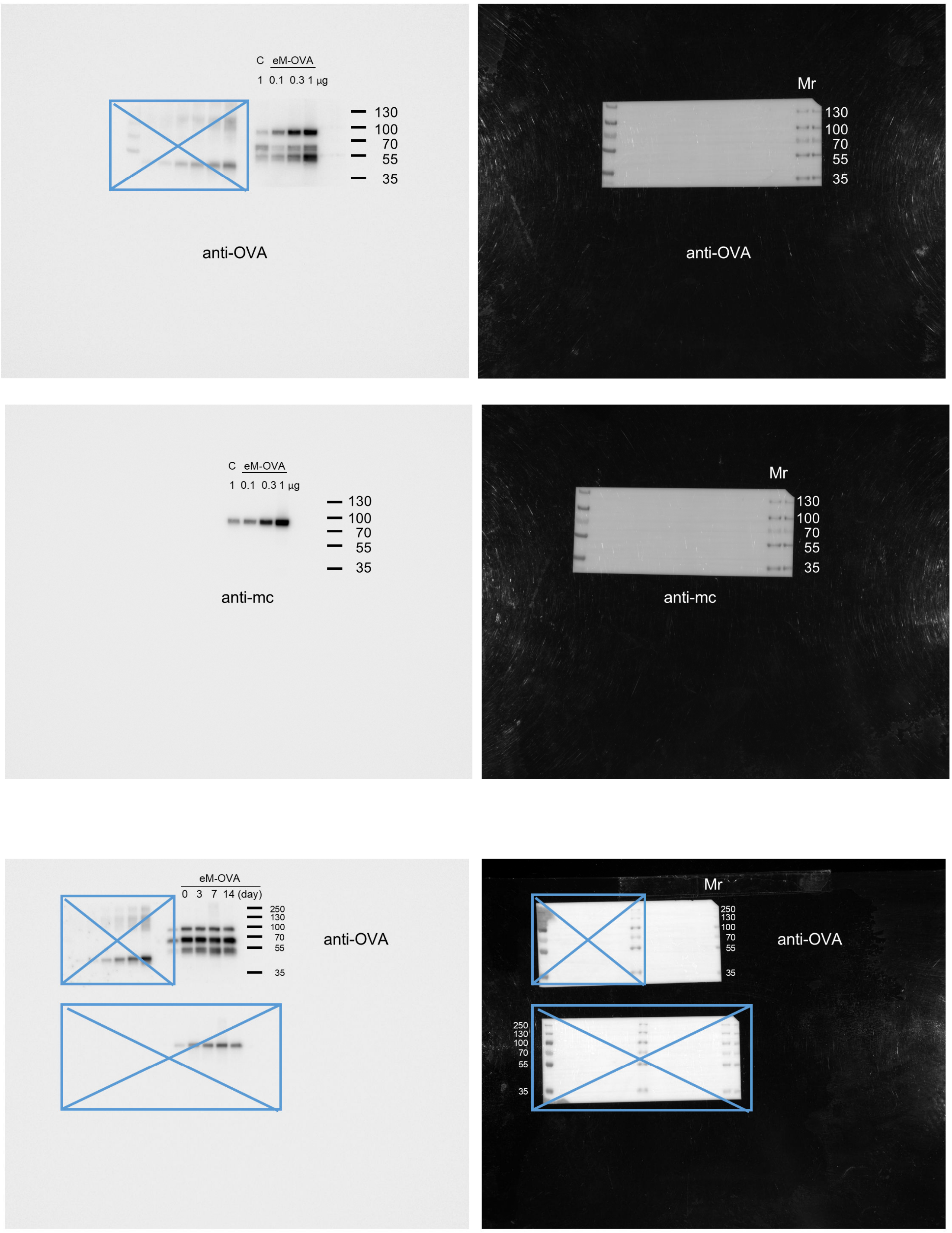

Figure 4, Source Data 1. The left images are the original chemiluminescence blots corresponding to Figure 4E and 4K. The right images are the corresponding colorimetric blots, showing the rainbow molecular weight markers employed. The membrane in the upper panel (anti-OVA) was stripped and reblotted using anti-mCherry antibody, presented in the middle panel.
