# Supplementary material for: Engineered migrasomes provide a robust and thermally stable vaccination platform
Source: eLife. 2025 Nov 13;13:RP97621. doi: 10.7554/eLife.97621 (PMC12614892; doi:10.7554/eLife.97621)
Supplement: Figure 5—source data 1. [file elife-97621-fig5-data1.zip › Figure 5-source data 1.pdf]

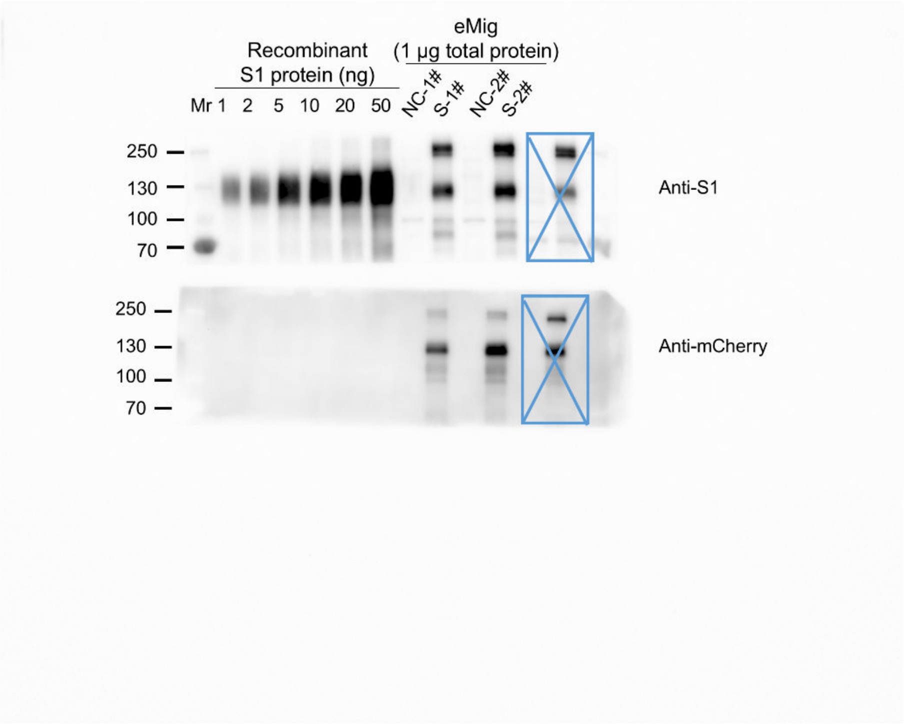

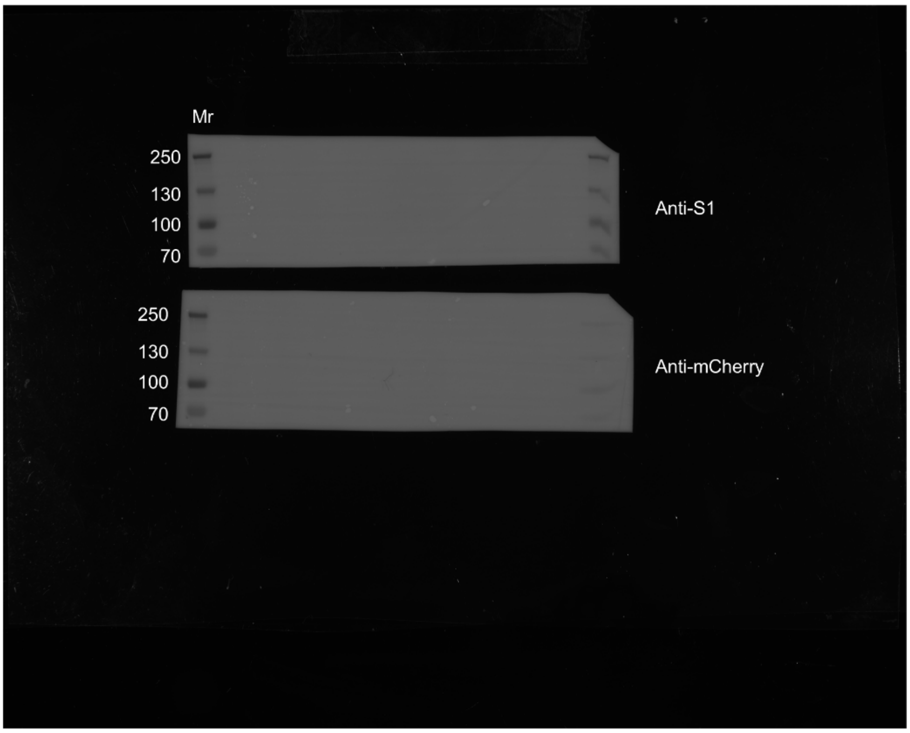

Figure 5, Source Data 1. The upper image is the original chemiluminescence blot corresponding to Figure 5B. The lower image is the corresponding colorimetric blot, showing the rainbow molecular weight markers employed.
